# Evidence that the Nijmegen breakage syndrome protein, an early sensor of double-strand DNA breaks (DSB), is involved in HIV-1 post-integration repair by recruiting the ataxia telangiectasia-mutated kinase in a process similar to, but distinct from, cellular DSB repair

**DOI:** 10.1186/1743-422X-5-11

**Published:** 2008-01-22

**Authors:** Johanna A Smith, Feng-Xiang Wang, Hui Zhang, Kou-Juey Wu, Kevin Jon Williams, René Daniel

**Affiliations:** 1 Division of Infectious Diseases – Center for Human Virology, Kimmel Cancer Center, Thomas Jefferson University, Philadelphia, PA, USA; 2 Institute of Biochemistry and Molecular Biology, National Yang-Ming University, Taipei, Taiwan; 3 Division of Endocrinology, Thomas Jefferson University, Philadelphia, USA; 4 Kimmel Cancer Center, Immunology Program, Thomas Jefferson University, Philadelphia, PA, USA; 5 704G Abramson Research Center, 3615 Civic Center Boulevard, Philadelphia, PA 19104, USA

## Abstract

Retroviral transduction involves integrase-dependent linkage of viral and host DNA that leaves an intermediate that requires post-integration repair (PIR). We and others proposed that PIR hijacks the host cell double-strand DNA break (DSB) repair pathways. Nevertheless, the geometry of retroviral DNA integration differs considerably from that of DSB repair and so the precise role of host-cell mechanisms in PIR remains unclear. In the current study, we found that the Nijmegen breakage syndrome 1 protein (NBS1), an early sensor of DSBs, associates with HIV-1 DNA, recruits the ataxia telangiectasia-mutated (ATM) kinase, promotes stable retroviral transduction, mediates efficient integration of viral DNA and blocks integrase-dependent apoptosis that can arise from unrepaired viral-host DNA linkages. Moreover, we demonstrate that the ATM kinase, recruited by NBS1, is itself required for efficient retroviral transduction. Surprisingly, recruitment of the ATR kinase, which in the context of DSB requires both NBS1 and ATM, proceeds independently of these two proteins. A model is proposed emphasizing similarities and differences between PIR and DSB repair. Differences between the pathways may eventually allow strategies to block PIR while still allowing DSB repair.

## Introduction

Post-integration repair (PIR) is an essential step in the retroviral lifecycle, and yet it remains incompletely understood. PIR occurs after the retroviral integrase has removed two nucleotides from the 3'-ends of viral DNA and then joined the newly exposed hydroxyl groups to staggered phosphates in complementary strands of the host chromosomal DNA, through non-blunt cleavage of host DNA in concert with the ligation reaction [1,2]. This initial integrase-mediated linkage between viral and host DNA produces an intermediate, in which the proviral DNA is flanked by short, single-stranded gaps in the host-cell DNA. PIR completes integration through four distinct steps: trimming the 2-bp flaps from the 5'-ends of the proviral DNA, filling in the single-stranded gaps that arose from the original staggered cleavage of host DNA, ligation of the trimmed 5' viral DNA ends to the filled-in host DNA strands, and reconstitution of appropriate chromatin structure at the integration site.

It has been proposed that that the virus exploits host-cell double-strand DNA break (DSB) repair pathways to complete the integration process, and initial evidence suggests that it involves the NHEJ (non-homologous end joining) pathway, as well as the ATM (ataxia telangiectasia mutated) and ATR (ATM and Rad3 related) kinases [3-9]. Nevertheless, several key issues remain. First, the earliest known sensor of DSBs, the Nijmegen breakage syndrome-1 protein (NBS1), has not been examined in the context of retroviral PIR. NBS1 is the crucial initiating component of the MRN complex, which comprises three proteins: MRE11 (meiotic recombination 11 homologue), a combined exo- and endo-nuclease [10]; RAD50, which binds DNA duplexes and may function as an anchor to hold the DNA ends together at a DSB [11]; and NBS1 itself. NBS1 associates with DSBs immediately after the DNA damage occurs [12] and recruits MRE11 and RAD50 [13,14]. In addition, NBS1 recruits the ATM kinase to DSB sites [15], and NBS1 [15] and ATM [16] are then both required to recruit the ATR kinase [16]. Activation of the ATM and ATR kinases allows them to phosphorylate several DNA repair and checkpoint proteins, including NBS1 itself [17-21]. Nijmegen breakage syndrome (NBS), which is caused by a hypomorphic mutation in the NBS1 gene, and ataxia telangiectasia (A-T), which is caused by mutations in the ATM gene, highlight the significance of NBS1 in DSB repair [22,23]. NBS and A-T cells exhibit similar DNA repair deficiencies, including hypersensitivity to γ-irradiation, which causes DSBs, and defective cell-cycle checkpoints that fail to arrest cell proliferation when unrepaired DSBs are present [21,24]. Because of the central role of NBS1 in DSB repair, we now hypothesize that this protein might initiate cellular responses leading to retroviral PIR as well.

The second key issue in understanding retroviral PIR concerns conflicting data in the literature about the roles of the ATM and ATR kinases. Although many publications demonstrated the participation of other NHEJ proteins in PIR [3,5,6,8,25,26], the precise roles for ATM and ATR remain less clear. For example, we reported only a minor function for the ATM protein, which became apparent mainly in the absence of other NHEJ components [5]. In contrast, some laboratories reported that ATM is required for efficient PIR even in the presence of NHEJ [8,27], whereas others reported efficient transduction even in the absence of ATM [28,29]. One explanation is that these discrepancies arose from the use of different immortalized cell lines in these studies. Therefore, in the current study we addressed the role of ATM in PIR in primary human cells.

Third, although a great deal is known about DSB repair, details of PIR have yet to be delineated. Retroviruses hijack numerous DSB repair proteins [3,5,6,8,25,26,30], but the geometry of retroviral integration differs considerably from DSB repair, which is limited to linking two blunt ends together. We now hypothesize that the two repair processes may crucially diverge. Initial supportive evidence comes from our recent finding that phosphorylation of the histone H2AX on its Ser 139 residue is crucial to DSB repair, but not for efficient PIR [31]. Importantly, differences between the two repair processes might allow strategies to inhibit PIR while still allowing NHEJ.

Therefore, we now sought to examine the presence, interactions, and function of several DSB repair proteins in retroviral PIR, namely, the initial DSB sensor NBS1 and the ATM and ATR kinases. Our comparisons of PIR with DSB repair continue to reveal fundamental similarities and differences.

### Experimental procedures

#### Primary human fibroblasts and lymphoid cell lines

All human cells were purchased from the Coriell Cell Repository (Camden, New Jersey): primary NBS fibroblast cells (deficient in the wild type NBS1 protein – GM07166) and matched control cells (GM04506); A-T primary fibroblasts (deficient in the ATM protein – GM02052) and matched controls (GM01661); EBV transformed NBS B-Lymphocytes (GM15818) and matched control EBV transformed cells (GM15817). All cells were maintained in RPMI-1640 medium in the presence of 10% fetal bovine serum (FBS), 5 × 10^-6^M 2-mercaptoethanol, non-essential amino acids, and 1% Pen/Strep.

#### HIV-1-based vectors

All VSV G-pseudotyped HIV-1-based vectors were prepared as described previously [3,32], and carried either a *lac*Z or EGFP reporter gene. A multiply attenuated vector (lacking the accessory proteins *vpr*, *nef*, *vpu *and *vif*) carrying the *lac*Z reporter is denoted as MAV [33].

#### Infections

Primary fibroblasts were plated at a density of 2 × 10^4 ^cells/well in 24-well plates, 10^5 ^cells/60-mm dish, or 3 × 10^5^/100-mm dish. B-Lymphocytes were plated at a density of 3 × 10^5^/ml in 24-well plates. Cells were infected the next day for 6 hours or overnight in the presence of 5 or 10 μg of DEAE-dextran per ml. Cultures were then assayed for reporter gene expression at multiple time points from two to seven days post-infection (dpi). Cells infected with *lac*Z-encoding viruses were stained overnight to detect β-galactosidase activity directly in dishes (Stratagene protocol) and blue cells were counted the following day. EGFP reporter gene expression was detected by flow cytometry. As a control to rule out non-specific effects of NBS1 deficiency on transient expression of *lac*Z, NBS1-deficient and control primary fibroblasts were plated at a density of 2 × 10^4 ^cells/well in a 24-well plate. The following day, cells were transfected with the *lac*Z plasmid, which encodes the *lac*Z reporter under control of the CMV promoter [32] using a ProFection Mammalian Calcium Chloride Transfection system (Promega). Cells were stained three days later for β-galactosidase activity (Stratagene protocol). To evaluate the effect of re-introduction of wild-type NBS1 on HIV-1 transduction, NBS1-deficient cells were plated at a density of 3 × 10^4 ^cells/well in a 24-well plates. The following day, cells were transfected with either an NBS1 expression plasmid or an empty vector, using the ProFection Mammalian Calcium Chloride Transfection system (Promega). One day post-transfection cells were infected with the HIV-1-based vector at a multiplicity of infection (m.o.i.) of 0.005. Cells were stained eight days later using a β-galactosidase assay as described above.

#### Chromatin Immunoprecipitation

**Figure 1:**
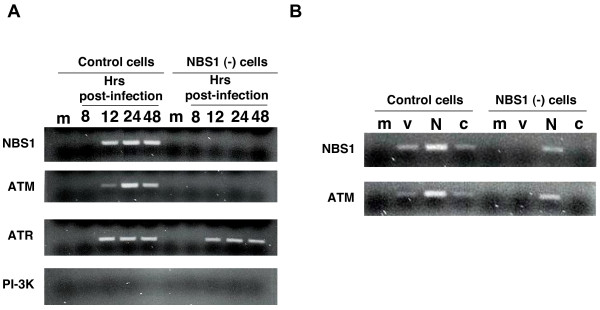
**NBS1 associates with viral DNA and is required for recruitment of ATM but not ATR**. **(A) **Chromatin immunoprecipitation of infected NBS1-deficient and control cells. To establish if NBS1, ATM, and/or ATR associate with viral DNA, normal and NBS1-deficient cells were infected with the HIV-1-based vector at an m.o.i. of 0.1 and chromatin immunoprecipitation was performed with anti-NBS1, anti-ATM and anti-ATR antibodies as described in the Experimental Procedures. m – mock, uninfected cells. The immunoprecipitating antibody is indicated on the left side of the photograph of the gel. **(B) **Chromatin immunoprecipitation of infected NBS1-deficient and control cells, which were transfected with the normal NBS1 gene. Control and NBS1-deficient cells were transfected with the NBS1-coding plasmid or an empty vector. 48 hrs post-transfection, cells were infected with the HIV-1-based vector at an m.o.i. of 0.1 and chromatin immunoprecipitation was performed 24 hrs later with anti-NBS1 and anti-ATM antibodies as described in the Experimental Procedures. m – uninfected cells, v – cells infected with the HIV-1-based vector, N – cells transfected with the normal NBS1 gene and infected with the HIV-1-based vector, c – cells transfected with the empty plasmid vector and infected with the HIC-1-based vector.

Chromatin Immunoprecipitation (ChIP) assays were performed as described previously [34]. 3 × 10^5 ^NBS1-deficient primary fibroblasts or control fibroblast cells were infected with our HIV-1-based vector (*lac*Z reporter) at m.o.i. 1. At the time points indicated, viral DNA and interacting proteins were cross-linked by the addition of formaldehyde (1% final concentration) to the cultures, which were then incubated for 30 min at room temperature. In the reconstitution experiment described in Figure 1B, cells were transfected with 50 μg of the NBS1 expression plasmid or the empty vector using the Lipofectamine™ 2000 reagent (Invitrogen, Cat no. 11668-027). 48 hrs after transfection, cells were infected with the HIV-1-based vector under conditions described above. Crosslinking was performed 24 hrs after addition of the virus. The cross-linking reaction was quenched with glycine (0.125 M final concentration). Plates were then washed with cold phosphate-buffered saline, and then scraped into phosphate-buffered saline containing protease inhibitors, and washed and lysed by addition of 0.5% Nonidet P-40, 5 mM PIPES, pH 8.0, 85 mM KCL and protease inhibitors. The intact nuclei were isolated by centrifugation at 5000 rpm at 4°C. Nuclei were then resuspended in a lysis buffer (1% SDS, 50 mM Tris-Cl, pH 8.1, 10 mM EDTA, protease inhibitors). Chromatin was sonicated to obtain DNA fragments of approximately 600 bp. Samples were subjected to centrifugation to remove debris and were precleared by shaking for 1 hr with salmon sperm DNA/protein A-agarose (Upstate, Temecula, CA, cat. no. 16–157), which were then removed and supernatants were diluted 10-fold with a dilution buffer (0.01% SDS, 1.1% Triton X-100, 1.2 mM EDTA, 16.7 mM Tris-Cl, pH 8.1, 167 mM NaCl, protease inhibitors). Chromatin fragments were immunoprecipitated overnight with antibodies against ATM (Santa Cruz Biotechnology, sc-15392), ATR (Santa Cruz Biotechnology, sc-1887), NBS1 (Santa Cruz Biotechnology, sc-8580), or, as a control, the irrelevant protein PI-3K 110δ (Santa Cruz Biotechnology, sc-55589). Protein-DNA-antibody complexes were isolated by the addition of salmon sperm DNA/protein A-agarose. After 1 hr, complexes were collected by centrifugation and washed three times with buffer (100 mM Tris, pH 8, 500 mM LiCl, 1% Nonidet P-40, 1% deoxycholic acid). Pellets were eluted from salmon sperm DNA/protein A-agarose with 50 mM NaHCO3, 1% SDS for 15 min at room temperature. Clarified samples were incubated with RNase and 5 M NaCl at 67°C for 4–5 hr to reverse cross-links and then precipitated overnight with ethanol. Following centrifugation, pellets were resuspended in proteinase K buffer and treated with proteinase K to digest residual proteins. After phenol/chloroform extraction, the DNA was precipitated with ethanol. Viral sequences in these fractions were detected by PCR using primers targeting the HIV-1 long terminal repeats: M667, 5'-GGC TAA CTA GGG AAC CCA CTG-3'; AA55, 5'-CTG CTA GAG ATT TTC CAC ACT GAC-3'[35]. The PCR reaction was done as follows: 94C for 5 min, then 30 cycles of 94C – 1 min, 55C – 1 min, 72C – 1 min. Final extension was run for 5 min at 72C. PCR products were resolved on an ethidium bromide-stained 2% agarose gel.

#### Alu-PCR

To detect and quantify fully integrated proviral DNA, a two-step nested PCR technique was conducted. Primary NBS1-deficient fibroblasts and control cells were infected with HIV-1-based vector (*lac*Z reporter) at m.o.i 1, m.o.i. 0.01, or mock infected. Three days post-infection genomic DNA was extracted (Promega kit A1120). First round of *Alu*-PCR employed a primer targeting the cellular *Alu *sequence 5'-GCC TCC CAA AGT GCT GGG ATT ACA G-3' as well as the M661 primer targeting the HIV-1 LTR/*gag *region, 5'-CCT GCG TCG AGA GAG CTC CTC TGG-3'. This initial amplification step used 150 ng of genomic DNA as template. Samples were subjected to 30 PCR cycles of 95C – 30 s, 60C – 45 s, and 72C – 5 min, and after the final round, samples were kept at 72°C for 10 min. Products of the first round were diluted 1/1,000 and used in the 30-cycle second round (nested) with viral LTR primers: 5'-GGA TTG TGC TAC AAG CTA GTA CC-3'; and 5'-TGA GGG ATC TCT AGT TAC CAG AGT-3'. Second-round PCR was cycled as follows: 95°C for 5 min; 30 cycles of 95°C for 40 s, 55°C for 45 s, 72°C for 60 s, and the last round was followed by 72°C for 10 min. PCR products from the second round were resolved by electophoresis on an agarose gel and subjected to Southern blotting with an HIV-1- LTR probe.

#### Statistics

Quantitative data are displayed as means ± standard deviations. Comparisons between two groups were performed using the two tailed Student t-test.

## Results

### The NBS1 protein is required for association of ATM, but not ATR, with viral DNA

Normal and NBS1-deficient primary human fibroblasts were infected with the pseudotyped HIV-1-based vector (*lac*Z reporter) at an m.o.i. of 0.1 and harvested at the indicated time points (Figure 1A). ChIP analysis was used to identify accumulation of NBS1, ATM, and ATR at sites of proviral DNA integration. Nuclear DNA and its associated proteins were crosslinked, immunoprecipitated with the indicated antibodies (anti-NBS1, ATM, or ATR), and associated viral DNA was amplified by PCR. Figure 1A shows an agarose gel of the amplified PCR products. In normal primary fibroblasts, the presence of viral DNA in NBS1, ATM, and ATR immunoprecipitates was first detected 12 hrs post-infection. In NBS1-deficient cells, however, we did not observe any association of viral DNA at any timepoint with NBS1, as expected, nor with ATM (Figure 1A). Surprisingly, NBS1 deficiency and the failure to recruit ATM did not block the association of viral DNA with ATR (Figure 1A, third row), even though NBS1 and ATM are each required for recruitment of ATR to DSB sites [15,16]. As a negative control, no viral DNA was detected in any sample immunoprecipitated with the irrelevant anti-PI-3K kinase antibody (Figure 1A, bottom row).

To verify that the failure of ATM association with viral DNA in NBS1-deficient cells arises specifically from the mutation in the NBS1 gene, rather than from some other difference between these and control cells, we performed NBS1 reconstitution studies. Normal and NBS1-deficient fibroblasts were transfected with either an expression plasmid for wild-type NBS1 or an empty vector [36]. Transfected cells were then infected with the HIV-1-based vector. The right half of Figure 1B shows ATM association with viral DNA in NBS1-deficient cells that were transfected with the NBS1 expression plasmid, but not in NBS1-deficient cells transfected with the empty vector, thereby confirming the essential role of NBS1. The NBS1 expression plasmid brought the amount of viral DNA associated with ATM to roughly the same level as in normal control cells (Figure 1B). Interestingly, overexpression of NBS1 in normal cells enhanced the association of viral DNA with ATM (Figure 1B, left half), suggesting that the NBS1 protein could be a limiting factor for ATM-mediated PIR even in normal cells. Taken together, our results demonstrate that NBS1 is required for association of ATM, but not ATR, with vector DNA.

### The NBS1 protein is required for efficient stable transduction of human fibroblasts by HIV-1-based vectors

**Figure 2:**
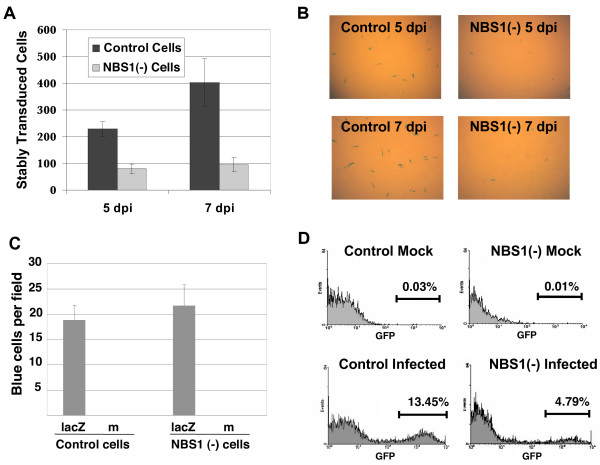
**NBS1 is required for efficient HIV-1 transduction of primary cells**. **(A) **NBS1-deficient fibroblasts (GM07166) and matched controls (GM04506) were infected with HIV-1-based vector carrying the *lac*Z reporter at an m.o.i. of 0.25. Five and seven days post infection (dpi) cells were stained using a β-galactosidase assay (Stratagene protocol) and transduced (blue) cells were counted under a light microscope the following day. Light grey – NBS1-deficient cells; dark grey – normal cells. The error bars represent standard deviation, p = 0.029 for 5 dpi and 0.021 for 7 dpi. **(B) **Light microscopic images from the same experiment as in A. **(C) **The effect of the NBS1 deficiency on expression of the *lac*Z marker. The NBS1-deficient and control cells were transfected with the *lac*Z plasmid and *lac*Z-expressing cells were counted three days post transfection. Six randomly selected fields were counted per each point. The error bars represent standard deviation. The differences were not statistically significant (p > 0.2) **(D) **Transduction with the HIV-1-based vector carrying the EGFP marker. NBS1-deficient and control fibroblasts were infected with the vector and transduced cells were counted by flow cytometry at multiple time points (2–7 dpi). Results from 7 dpi are shown. Histograms of mock infected cells (top) and cells infected at an m.o.i. of 0.1 (bottom) are shown. As seen in the gated regions, 13.45% of control fibroblasts were stably transduced, whereas transduction of NBS fibroblasts was only 4.79%.

Given our finding of NBS1 association with DNA of the HIV-1-based vector, we sought to determine its role in the life-cycle of the HIV-1-based vectors. Normal and NBS1-deficient primary fibroblasts were infected with the HIV-based vector carrying the *lac*Z reporter at an m.o.i. of 0.025, and the infected cells were counted by staining for β-galactosidase activity at late timepoints, indicative of stable retroviral transduction (5–7 days post-infection, dpi). Of note, we observed that the NBS1-deficient primary fibroblasts in this study grew at a rate close to that of normal cells and exhibited the same plating efficiency as normal cells. As shown in Figure 2A, the infection efficiency of NBS1-deficient fibroblasts was only 35% of that of the control cells at 5 dpi and decreased to 24% of the control value at 7 dpi. Figure 2B shows typical microscopic images used to generate the quantitative data in Figure 2A. To verify that NBS1 deficiency does not directly affect the *lac*Z reporter, control and NBS1-deficient cells were transfected with the non-viral *lac*Z plasmid, and β-galactosidase activity was quantified in cells 3 days later by staining. As shown in Figure 2C, NBS1 deficiency did not alter CMV-driven *lac*Z expression.

To test whether the transduction deficiency of NBS1-deficient cells can be observed using another reporter gene, control and NBS1-deficient primary human fibroblasts were infected with an HIV-1-based vector carrying the EGFP reporter [3]. At an m.o.i. of 0.1, 13.45% of control cells expressed the reporter gene whereas EGFP expression was detected in only about one third as many NBS1-deficient fibroblasts (4.79%, Figure 2D). Based on the results of these different assays, we conclude that NBS1 deficiency substantially decreases stable retroviral transduction of primary human fibroblasts. We note that a drop of transduction efficiency of NBS1-deficient cells was noted previously (about two fold), but the data were not further analyzed [37].

### The transduction deficiency of the NBS1-deficient cells can be rescued by expression of normal NBS1

**Figure 3:**
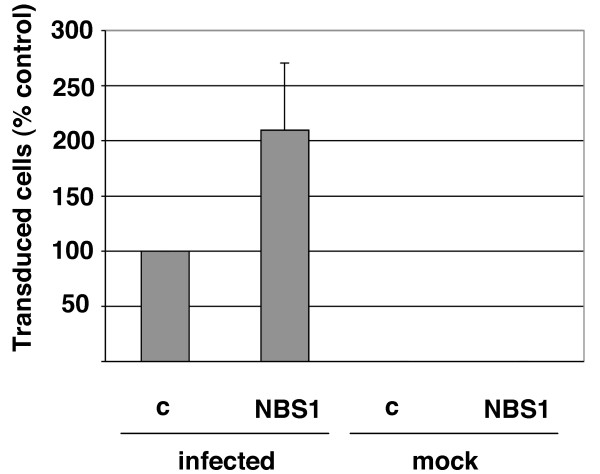
**Reintroduction of normal NBS1 cDNA into NBS1-deficient cells restores HIV-1 transduction efficiency**. NBS1-deficient cells were transfected with a plasmid encoding normal NBS1 cDNA or an empty vector plasmid. One day post-transfection, cells were infected with the HIV-1-based vector carrying the *lac*Z reporter. Cells were then stained eight days post-infection using a β-galactosidase assay and transduced (blue) cells were counted. c – control (cells transfected) with the empty vector, NBS1 – cells transfected with the plasmid carrying the normal NBS1 gene. The error bars represent standard deviation, p = 0.037.

The transduction deficiency of NBS1-deficient cells could be conceivably due to an additional mutation gained by these cells, instead of the NBS1 mutation. To test this hypothesis, NBS1-deficient fibroblasts were transfected with the expression plasmid for wild-type NBS1 or the empty control vector [36]. Transfected cells were then infected with the *lac*Z-carrying HIV-1-based vector. As shown in the Figure 3, transduction efficiency of NBS1-deficient cells reconstituted with the NBS1 expression plasmid was more than twice the level in cells that received the empty vector. Thus, the deficiency in retroviral transduction in NBS1-deficient cells arises directly from the mutation in the NBS1 gene.

### The NBS1 protein is required for efficient transduction of human lymphoid cells by HIV-1-based vectors

**Figure 4:**
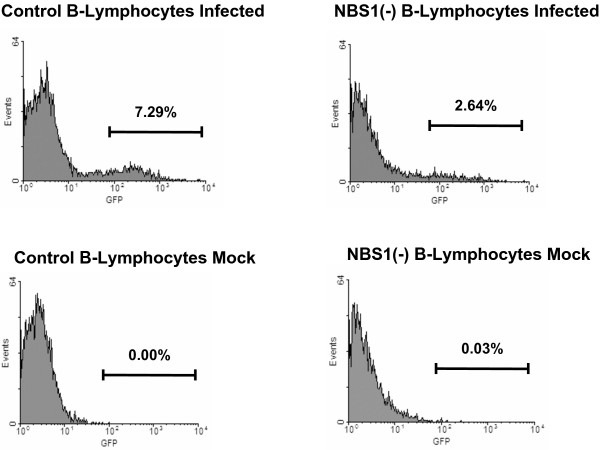
**NBS1 is required for efficient HIV-1 transduction of lymphoid cells**. EBV-transformed NBS1-deficient B-lymphoid cells (GM15818) and matched control EBV-transformed cells (GM15817) were infected with the HIV-1-based vector carrying the EGFP reporter and then assayed 7 dpi by FACS to quantify reporter gene expression. At an m.o.i. of 0.1, control lymphocytes were infected at a rate of 7.29%, while only 2.64% of NBS lymphocytes were infected.

To determine if retroviral transduction depends on NBS1 in cells other than primary human fibroblasts, EBV-transformed B-lymphoid cells from normal and NBS subjects were infected with the HIV-1-based vector carrying the EGFP marker. Transduction efficiency was measured 7 dpi by flow cytometry. At an m.o.i. of 0.1, 7.29% of control cells were infected, while NBS EBV-transformed B-lymphocytes were infected at approximately one-third the rate (2.64%, Figure 4). Thus, similar to NBS1-deficient fibroblasts, NBS1-deficient B-lymphoid cells exhibit substantially decreased transduction efficiency by HIV-1-based vectors, indicating that efficient HIV-1 transduction requires NBS1 in other cell types as well.

### The transduction deficiency of NBS cells does not result from a defect in vpr-mediated cell-cycle arrest

**Figure 5:**
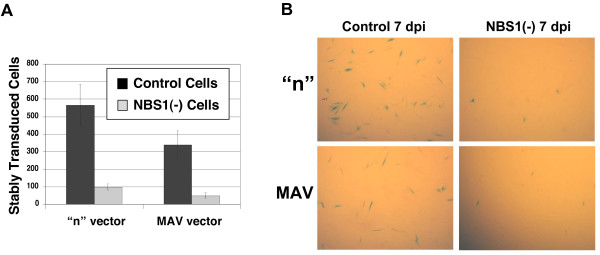
**NBS1 facilitates HIV-1 transduction independently of the vpr gene**. Cells were infected with either the normal ("n") or MAV vector at an m.o.i. of 0.1, and then stained overnight using a β-galactosidase assay at seven dpi. **(A) **Stably transduced cells per dish. **(B) **Pictures under the light microscope from the same experiment.

An HIV-1 accessory gene, *vpr*, was reported to induce G2 cell-cycle arrest by triggering the ATR-dependent checkpoint cascade [38]. Hypothetically, *vpr *could increase HIV-1 transduction by inducing the growth arrest, thereby giving the cell additional time to complete PIR. Since NBS1 is involved in cell-cycle checkpoint activation as well [39], it is conceivable that NBS1 deficiency could result in loss of *vpr*-induced growth arrest and it this way lead to reduced HIV-1 transduction. If this were the case, then the NBS1 deficiency should not affect HIV-1 transduction in the absence of *vpr*. To test this hypothesis, we infected control and NBS1-deficient fibroblasts with a multiply attenuated HIV-1-based vector (MAV) that is missing the *vpr *gene. Separate cells were infected in parallel with the original non-attenuated HIV-1-based vector, which contains *vpr*. Figure 5 shows that control cells were infected with both vectors at approximately 6-fold higher rates than NBS1-deficient cells. Because MAV does not contain the viral *vpr *gene, reduced transduction of NBS1-deficient cells cannot be attributed to a lack of *vpr*-mediated cell cycle arrest.

### The NBS1 protein is required for efficient joining of viral DNA to host cell DNA, but does not affect other steps in the HIV-1 life cycle

**Figure 6:**
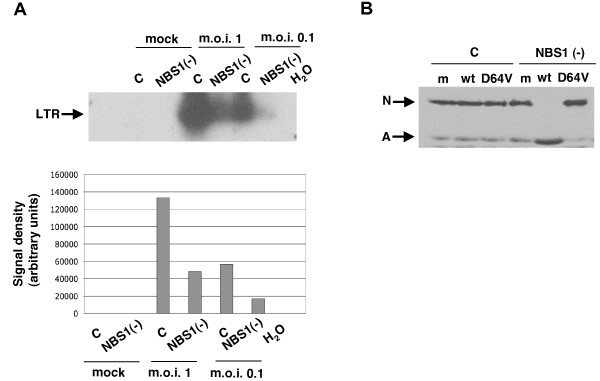
**NBS1 is required to efficiently complete the integration of viral DNA and to avoid integrase-dependent apoptosis**. **(A) **Completed integration in NBS1-deficient vs. normal control cells. Alu-PCR was performed to detect viral-host DNA junctions. In this nested PCR technique, genomic DNA was extracted from HIV-1-infected NBS1-deficient and control cells at 3 dpi. The first round of PCR was performed with one primer targeting the virus LTR region, and the other primer targeting cellular *Alu *sequences. The second round utilized two LTR primers. Top – the amplified viral sequences were detected by southern blotting. Bottom – Southern was quantified by densitometry. **(B) **PARP cleavage in infected cells. Normal and NBS1-deficient cells were infected as described in the Experimental Procedures. Two days post-infection, cells were harvested, lysed and cell lysates subjected to western blotting with an anti-PARP antibody. wt – cells infected with an integration-competent HIV-1-based vector, D64V – cells infected with the vector carrying the D64V mutation in the integrase protein.

To determine which step of the vector life-cycle involves NBS1, we infected primary NBS1 and control fibroblasts with the HIV-1-based vector carrying the *lac*Z reporter and analyzed viral DNA synthesis, nuclear import of viral DNA, and completed DNA joining events by *Alu*-PCR at 3 dpi. NBS1 deficiency did not measurably decrease viral DNA synthesis or nuclear import as measured by formation of 2-LTR circles (data not shown), the latter finding being consistent with Kilzer *et al*. Importantly, the number of completed joining events was reduced by approximately two-thirds in NBS1-deficient fibroblasts relative to the control cells (Figure 6A). Thus, NBS1 is involved in the joining of viral to host DNA.

### Retroviral infection triggers apoptosis of NBS1-deficient cells in an integrase-dependent manner

The decreased amount of viral DNA that is joined to host cell DNA in NBS1-deficient cells presumably results from a failure of PIR. As a theoretical alternative, the NBS1 protein might be required for the initial integrase-mediated joining reaction. To distinguish between these possibilities, we took advantage of the fact that failure of PIR after integrase-mediated joining of viral and host DNA in other contexts triggers apoptosis through activation of cell-cycle checkpoint proteins by the unrepaired intermediate, leading to a loss of infected cells from the population [3,5,6,8,25,26]. Thus, normal and NBS1-deficient fibroblasts were infected at a high m.o.i. (4.0) with an integration-competent HIV-1-based vector or a vector carrying the enzymatically inactive D64V mutation in the integrase protein. Cells were further analyzed by Western blotting for the presence of the 85-kDa PARP fragment, an apoptotic marker generated by caspase-mediated cleavage of the PARP protein [40]. As shown in the Figure 6B, only one infection condition stimulated PARP cleavage, namely, infection of NBS1-deficient cells with the integrase-competent HIV-1-based vector. Neither cell type underwent apoptosis after infection with the integrase-deficient virus, and neither viral construct induced PARP cleavage in the normal cells. Thus, HIV-1 infection induces apoptosis of NBS1-deficient cells in an integrase-dependent manner. This finding is consistent with the failure of PIR rather than a defect in the initial integrase-mediated joining.

### The ATM kinase is required for efficient HIV-1 transduction of primary human cells

**Figure 7:**
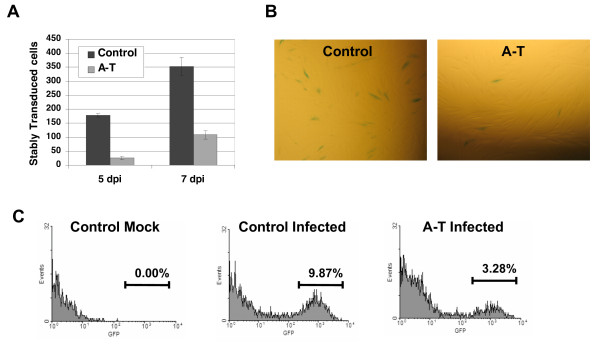
**ATM is required for efficient transduction of primary fibroblasts**. **(A) **HIV-1 transduction of the *lac*Z marker as measured by detecting *lac*Z reporter activity in infected A-T fibroblast and control fibroblast cells. Cells were infected with an HIV-1-based vector carrying the *lac*Z reporter at an m.o.i. of 0.3. Infected cells were stained overnight using a β-galactosidase assay at five and seven dpi and transduced cells counted. **(B) **Light microscopic images from the same experiment as in A, taken 4 dpi. **(C) **A-T and control fibroblast infections with the HIV based vector carrying the EGFP marker. Cells were evaluated by flow cytometry at multiple time points (2–7 dpi). Results from 7 dpi are shown. Histograms of mock-infected control cells (left), control cells infected at m.o.i. of 0.1 (middle), and infected A-T cells are shown. The error bars represent standard deviation, p = 0.0014 for 5 dpi and 0.027 for 7 dpi.

As noted in the Introduction, ATM was proposed as an essential host factor for PIR, but the literature contains conflicting data [5,8,27,28], possibly owing to the use of different transformed cell lines by different laboratories. Moreover, DSB requires both NBS1 [15] and ATM [16] for the recruitment of ATR, yet our ChIP studies demonstrated that ATR robustly localizes to sites of PIR without either of these proteins (Figure 1A, third row). Thus, cells specifically deficient in ATM, despite their defect in DSB repair [21,24], would still exhibit localization of both NBS1 and ATR to sites of viral integration, and it is possible that these proteins would then mediate PIR independently of ATM. To test this possibility in non-transformed cells, we infected normal and ATM-deficient (A-T) primary human fibroblasts with our HIV-1-based vectors. ATM-deficient cells reproducibly demonstrated a decrease of transduction efficiency by 60–80% compared to normal cells, regardless of the readout method or the transduced reporter (Fig 7). These results agree with Lau *et al*. [8] and support the hypothesis that ATM is required for efficient PIR in primary human cells, despite the independent recruitment of ATR (Figure 1A).

## Discussion

In this study, we demonstrated that NBS1, an early sensor of DSBs, associates with viral DNA, is required for the association of ATM – but not ATR – with viral DNA, mediates efficient integration of viral DNA, promotes stable retroviral transduction, and blocks integrase-dependent apoptosis that can arise from unrepaired viral-host linkages. These data support a key role for the NBS1 protein in PIR. We and others proposed that retroviral PIR employs the NHEJ pathway, including the ATM and ATR kinases [3-9]. Our current results extend that work, by demonstrating the dependence of PIR on NBS1, an interaction between NBS1 and ATM, and a dependence on ATM for PIR in primary, non-transformed cells. All of these features are shared with cellular DSB repair.

Nevertheless, the integration intermediate structurally differs from a DSB (see the Introduction), and so we now revised our model to include the concept that the two repair processes may diverge in key aspects. Initial evidence that PIR uses somewhat different cellular machinery than DSB repair came from our recent study where we observed that phosphorylation of the histone H2AX isoform, which is mediated by both the ATM and ATR kinases and is required for DSB repair, appears to be dispensable for PIR, although it can be detected at the integration sites [31]. Importantly, our current results establish the surprising finding that recruitment of ATR, which in the context of DSB requires both NBS1 and ATM, proceeds independently of these two proteins. In this context, we note that some HIV-1 transduction occurs even in the absence of normal NBS1 or ATM (see Results section). It is possible that this residual transduction is mediated by ATR.

One possible explanation for the difference in ATR recruitment in PIR vs. DSB repair could be that the single-stranded DNA gaps, which flank the integration site, are sufficient to recruit the ATR protein. In contrast, MRN-dependent processing of DSBs, which may generate single-stranded DNA through the nuclease activity of MRE11, appears necessary for accumulation of ATR at the DSB sites [16]. Additional differences between these DNA repair processes may exist, and might guide the development of therapeutic strategies to selectively inhibit PIR without blocking DSB repair.

**Figure 8:**
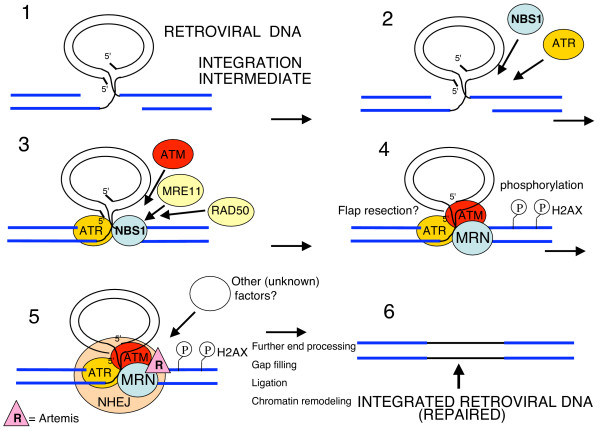
**Model for the role of NBS1 in post-integration repair**. Integrase catalyzes formation of the integration intermediate **(1)**. NBS1 and ATR are recruited independently to the integration sites **(2)**. NBS1 then recruits MRE11, RAD50 and ATM. **(3)**. The 5'-end DNA flaps of the viral DNA are trimmed, possibly by MRN. ATM phosphorylates H2AX. However, H2AX phosphorylation is not required for post-integration repair. **(4)**. We speculate that Artemis and NHEJ proteins are recruited to the integration site **(5)**. These proteins, and likely other factors, then mediate the other steps of post integration repair, which require possibly further end processing, gap filling, ligation and chromatin remodeling **(6)**.

Our discovery of a crucial role for NBS1 in PIR opens several possibilities with regards to the molecular mechanism of PIR. First, the simplest model is that NBS1 acts primarily through its recruitment of the ATM kinase to integration sites, as suggested by our results. ATM, in turn, may phosphorylate other proteins at integration sites and thus regulate their activity. Interestingly, it was recently shown that ATM phosphorylates the DNA repair protein Artemis, and ATM is required for Artemis-dependent processing of damaged ends of DNA [41]. Artemis is a critical component of the cellular non-homologous end joining (NHEJ) DNA repair pathway and these data thus provide a link between the ATM kinase and NHEJ pathway. We and others have presented extensive evidence indicating that NHEJ is involved in PIR [3,5-8]. One could thus imagine that NBS1 exerts its effect on PIR by regulating the ATM-Artemis-NHEJ pathway. However, NBS1 is also a component of the MRN complex, and recruits the MRE11 nuclease of this complex to the sites of DNA breaks [13,14]. The process of PIR involves trimming of 5'-viral DNA ends prior to joining of viral and host DNA ends. An intriguing role for MRE11 in the MRN complex would be trimming of these short flaps of viral DNA (Figure 8).

As we and others have suggested, cellular co-factors constitute an attractive target for anti-HIV-1 therapy, since development of resistance against inhibitors of these proteins is unlikely [6,8,9,27,42]. NBS1 and its interactive partners, being such co-factors, are thus potential targets for anti-HIV-1 therapeutics, particularly at steps where PIR differs from DSB repair.

## Competing interests

The author(s) declare that they have no competing interests.

## Authors' contributions

JAS carried out the HIV-1 transduction experiments and β-galactosidase and EGFP assays. FW carried out the *Alu*-PCR assay. KW provided the NBS1 and control vectors and participated in designing the NBS1 reconstitution experiment and revising the manuscript. HZ and KJW extensively participated in drafting the manuscript and experimental design. RD conceived of the study, carried out the chromatin immunoprecipitations and western blotting experiments and wrote the manuscript. All authors read and approved the final manuscript.
